# Disease Control Priorities Third Edition Is Published: A Theory of Change Is Needed for Translating Evidence to Health Policy

**DOI:** 10.15171/ijhpm.2018.60

**Published:** 2018-06-30

**Authors:** Ole F. Norheim

**Affiliations:** Department of Global Public Health and Primary Care, University of Bergen, Bergen, Norway.

**Keywords:** Theory of Change, Priority Setting in Health, Economic Evaluation, Equity

## Abstract

How can evidence from economic evaluations of the type the Disease Control Priorities project have synthesized be translated to better priority setting? This evidence provides insights into how investing in health, particularly though priority interventions and expanded access to health insurance and prepaid care, can not only save lives but also help alleviate poverty and provide financial risk protection. The article discusses some of the relevant factors needed to develop a Theory of Change for translating economic evidence to better priority setting within countries, and proposes some key strategic choices that are necessary to achieve the desired outputs and outcomes.

The Disease Control Priorities project aims to promote and support the use of economic evaluation for priority setting at both global and national levels. The first edition, *DCP1*, was published by the World Bank in 1993, as a companion volume to the World Bank report “Investing in Health” commissioned by chief economist Larry Summer and written by a team that included a young doctor and health economist Chris Murray, with economist Dean Jamison as senior lead author.^1,2^ In a famous interview in The New Yorker, Bill Gates told the story of how Investing in Health influenced him on how to spend his wealth when moving into philanthropy.^3^ This small report has been hugely influential though international actors, also beyond the Bill and Melinda Gates Foundation.^4^

The report provided a convincing analysis that investing in health could yield substantial economic returns. Healthcare is not only an expenditure for the ministries of finance. If resources are invested wisely, people will live longer and have more productive lives. Healthy children learn better, malaria prevention reduces absenteeism and increases productivity, and public finance protects against catastrophic health expenditures.

A key message of DCP1 was that resource allocation within the health sector should aim at health maximization, and the tool to identify which interventions and policies to invest in is cost-effectiveness analysis. By modeling costs per disability-adjusted life year (DALY) averted, the most efficient ranking of services can be determined. The report also proposed that countries should invest in an essential package of public health interventions and a package of essential clinical services that would yield the maximum health benefit.^1^ DCP2 was published in 2006 with updated economic analyses and a more developed emphasis on service delivery platforms: at what level of the health system are policies and services most effective?^5^

The recommendations from the Disease Control Priorities Project have also been met with considerable resistance and criticism. While the World Health Organization (WHO) and the World Bank early on championed cost-effectiveness as a key criterion for global and national resource allocation,^2,6^ philosophers, ethicists and others in the social justice tradition have argued that this approach is insensitive to the distributional aspects of priority setting.^7-9^ Equity also matters. Other have discussed whether a reliance on cost-effectiveness alone is compatible with a human rights approach to health and healthcare.^10^

Some even argued that all substantive values underlying priority setting, and especially cost-effectiveness, are so contested that they should be replaced by a fair and legitimate process.^11^ Others hold that both process and substantive judgments are important.^7,8,12^ The broader democratic processes include better governance for health and public participation. Governments and other relevant institutions can be held accountable for ensuring that proper participatory processes are in place.^13,14^ Yet, few countries have really succeeded in going beyond technocratic approaches to inclusive priority setting. The “political determinants” of health, barriers to global governance such as power structures and unfair trade agreements, can also hamper change.^15^ Health policy needs to go beyond cost-effectiveness, to set priorities with respect to the worse off (in terms of health and poverty) and financial risk protection – and though robust processes in each country.^16^

## Disease Control Priorities, Third Edition

In December 2017, the ninth and final volume of DCP3 was published and launched in London.^17,18^ The volume, and its companion paper in the Lancet, summarizes recommended essential Universal Health Coverage (UHC) packages for low- and lower-middle-income countries for all areas of health: intersectoral policies, laws and regulations, financing, public health, as well as health services relating to surgery, cancer, noncommunicable diseases, injuries, mental health, adolescent health, reproductive, maternal and child health, and infectious diseases. These can be progressively implemented to speed up the move towards UHC as fulfilment of the Sustainable Development Goals for health (SDG3) and poverty (SDG1). Recognizing that the costs of such comprehensive packages would not, for some countries, find room within the fiscal space for health, DCP3 also recommends a highest priority package that could be implemented first, and at less cost.^18^

## Translating Evidence Into Better Priority Setting

DCP3 in many ways responds to earlier criticisms and widens the frame for discussion of health policies and priorities by addressing the different needs of countries at different stages in the development of their health systems. DCP3 also draws attention to equity and the catastrophically impoverishing effects that paying out of pocket for health services can have on poor families. This analysis, using data on private health expenditures, provides insights into how investing in health, particularly in expanded access to health insurance and prepaid care, can not only save lives but also help alleviate poverty and bolster financial security.

Several countries have expressed interest in using DCP3 evidence for better priority setting, and concrete projects have been initiated in Ethiopia, Afghanistan, Iran, and other countries in the Eastern Mediterranean Region (WHO EMRO).^19^ The question is how this can best be done. A first step would be to formulate and discuss a Theory of Change.

## Towards a Theory of Change for Better National Priority Setting

To translate evidence into policy for better priority setting, a complex process in itself, several factors need to be in place, work together, and lead to the desired outcomes. Making explicit the underlying Theory of Change is one approach recommended to clarify what is needed for achieving goals of this type. Theory of Change was developed within the tradition of theory-driven evaluation. Many organizations have used Theory of Change to improve programme implementation.^20^

Definitions of Theory of Change vary greatly. Breuer et al define Theory of Change as an approach which describes how a programme brings about specific long-term outcomes through a logical sequence of intermediate outcomes.^20,21^ A Theory of Change is often developed using a backward mapping approach which starts with the long-, medium-, and short-term outcomes and then maps the required process of change.

According to Vogel, the elements typically include outcomes, assumptions about what needs to be in place for change to occur, a description of contextual factors, beneficiaries, research evidence supporting the Theory of Change, actors in the context, their sphere of influence, strategic choices and interventions, timelines, and indicators.^20^

In what follows I sketch and discuss some of the relevant factors needed to develop a Theory of Change for translating economic evidence of the type DCP has generated to better priority setting within countries. I do not aim to develop a full-fledged Theory of Change fit for this purpose, but rather start a debate on what we need to understand before embarking on such an endeavor.

First, we need to define what we mean by translating DCP evidence into better priority setting. The Disease Control Priorities Project aimed to promote and support the use of economic evaluation for priority setting at both global and national levels. In DCP3, the aim has been further clarified by including a concern for equity – fair distribution – and financial risk protection. In the next phase of DCP, more emphasis is needed at national levels. One definition of better priority setting within countries would include three goals: to improve population health, not only the average level of health but also its distribution, with financial protection. Since we are in the era of SDGs and UHC, we may even formulate the most ambitious long-term outcome of priority setting as “all people receiving essential quality health services that meet their needs without being exposed to financial hardship in paying for the services.”^16,22^

## Better Priority Setting as a Long-term Outcome for Countries

Better national priority setting goes beyond what DCP can achieve. Yet, it is useful to describe a fair system in which economic evidence has a key role. A fair system for evidence-based priority setting in the health sector would include the following three elements: (*a*) Clear priorities: a reasonably well defined essential package of essential health services or publicly available descriptions of high-priority services to which people are entitled and information about how these services are to be financed; (*b*) Publicity: the package or range of high priority services is known by policy makers, actors in the health system, and citizens; and (*c*) Institutionalization: the process of priority setting is institutionalized and supported by the political system, is open and transparent, protected by legal regulation, linked to financing mechanism, and enjoys reasonable public support.

Clearly, such aims would be too ambitious and hard to measure by a set of indicators for a more modest Theory of Change. Figure summarises some elements of a Theory of Change for DCP country translation work.

**Figure:**
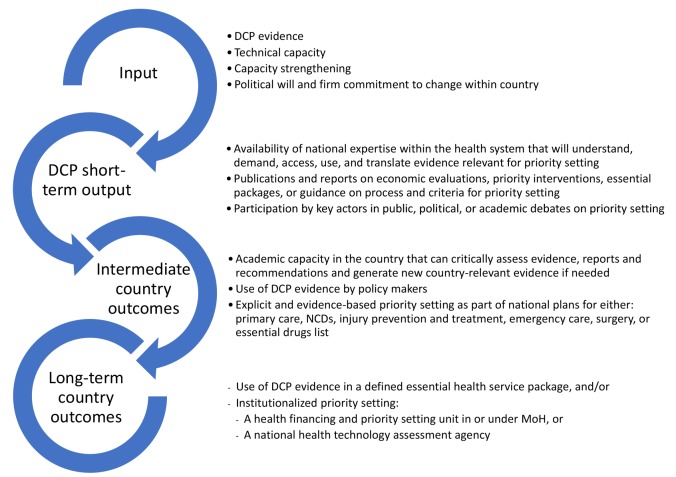
Elements of a Theory of Change for DCP Country Translation Work. Abbreviations: DCP, Disease Control Priorities; NCDs, non-communicable diseases; MoH, Ministry of Health.

## Long-term Country Outcomes for DCP

There are several of the above elements that go far beyond what a project such a DCP could achieve. I therefore propose the following alternative, but not mutually exclusive long-term outcomes:

– Use of DCP evidence in a defined essential health service package for
a. the whole health system, or for
b. delivery platforms such as primary care, public health, or intersectoral programs, or for
c. programs such as reproductive, maternal, neonatal, and child health services, non-communicable diseases (NCDs) services, or emergency care, or
– Institutionalized priority setting: eg, establishment of a health financing and priority setting unit in Ministry of Health (MoH) or under MOH (such as in a national Public Health Institute), or
– Establishment of a national health technology assessment (HTA) agency (under the MoH or independent from it).

Although creating institutions is the responsibility of countries, DCP could help and support institutionalizing priority setting. For some countries, these long-term outcomes could be achieved within a 5-year period, for others, the time horizon would be longer.

## Medium-term Country Outcomes for DCP

Realistic and medium-term outcomes for DCP country translation work could include the following:

– Availability of national expertise within the health system that will understand, demand, access, use, and translate evidence relevant for priority setting, and
– Explicit and evidence-based priority setting as part of comprehensive strategic plans to achieve UHC, or
– Explicit and evidence-based priority setting as part of national plans for primary care, NCDs, injury prevention and treatment, emergency care, surgery, or essential drugs list, etc, or
– Use of DCP evidence (and economic evaluations from other sources) by policy makers to make priority setting decisions.

## Options for Defining Short-term DCP Outputs

Short-term outputs that could be achieved, and that are preconditions for achieving the long-term outcomes, could include: (*a*) academic capacity in the country that can critically assess evidence, reports and recommendations and generate new country-relevant evidence if needed; (*b*) publications and reports on economic evaluation, priority interventions, essential packages, or guidance on process and criteria for priority setting; and (*c*) participation by key actors in public, political, or academic debates on priority setting.

## Preconditions for Better Priority Setting

What needs to be in place for the desired change to occur? In my view, at least five elements must be in place. First, there must be available timely, accurate and accessible evidence on costs and outcomes relevant for health systems strengthening, such as policy instruments, cross-sectoral public health actions, and clinical interventions tailored to delivery platforms or disease programs of relevance to governments and other actors that want to set priorities. Although incomplete, and not always contextualized to all national settings, DCP3 evidence is published and available, and is a good starting point for work on translation. More work needs to be done to make it even more available through an interactive web site with easily downloadable data and results. Evidence needs to be more accessible and packaged in more user-friendly formats. Second, in each country that embarks on this process, there must be political will to initiate work on translating DCP evidence to policy. Such support should be available from the highest levels of the MoH, given the political nature of priority setting. Third, a minimum of interest, expertise and capacity for initiating work on translating DCP evidence into policy is necessary to start the process. This is more likely to be more true in lower-middle-income countries than in low-income countries, but most countries today have some expertise within the bureaucracy that could understand and initiate the process. Fourth, if national expertise is incomplete, there must be available technical support and capacity for training in the initial phase. In South East Asia, Thailand’s Health Intervention and Technology Assessment Program (HITAP), supported by the International Decision Support Initiative (iDSI), serves as a hub for providing such expertise to neighboring countries.^23^ DCP and WHO-CHOICE also provide such support.^24^ Finally, a program for translating evidence to policy in low-income countries and lower-middle-income countries requires funding, from national sources and often from external sources as well.

## Contextual Factors

A careful assessment of contextual factors is also needed, although not all of the following can be influenced by projects such as DCP. Every country is special, and there are significant differences between low-income and lower-middle-income countries. Many of them are expanding their system towards the goal of UHC and have a unique opportunity to create new procedural mechanisms and institutions for better priority setting.

Yet, with respect to the prospect of implementing better systems for priority setting, many of the countries face substantial challenges. All countries have scarce resources for health. Low-income countries often rely on substantial external funding and donors have extensive influence on national priorities. There are also in many countries few people with expertise to understand, demand, access, use, and translate evidence relevant for priority setting. Another obstacle may be lack of political will. Explicit, accountable priority setting is not always preferable to implicit rationing, as may be easily understood if we adopt a political economy perspective on the hard choices they face.^25-27^ Under extreme resource scarcity, priority setting is after all about life and death, about distributing benefits and burdens, about winners and losers. Responsible policy makers also face considerable pressure from political elites and a growing middle class with political voice and influence. Priorities made often reflect these kinds of political constraints.

The professional organizations are often weak, with little influence on the professional direction and development of the health system. Another common characteristic is an unregulated private sector leading to overutilization, overpricing, and inefficiencies. Low salaries force many working in the public sector to also work elsewhere. Health systems are more often than not fragmented, mostly developed for providing basic prevention and health promotion and acute care for infectious diseases, child and maternal health. Low-income countries often rely on vertical programs that have proven efficient in earlier phases, but are ill equipped to handle growing needs for NCDs services. Lower-middle-income countries are in the midst of that transition toward integrated, comprehensive services for chronic conditions.

As for planning and budgeting, there is often inertia, and budgeting processes are typically based on previous allocations that are hard to shift. Add to this complex health financing mechanisms with multiple sources of funding, high out of pocket expenditures and inadequate prepayment mechanisms.^22^ Better priority setting for which services should be included under public finance presuppose that a larger share of total health expenditures is shifted to pooled funds. Since there are often weak mechanisms for pooling of resources and collection of tax revenue, and limited health insurance (often only for select groups such as civil servants), decision-makers’ control of funds is much more limited than in single payer systems such as the National Health Service (NHS) in the United Kingdom or in the Nordic countries. Another real challenge is to reach and convince strong ministers and ministries of finance that they should invest in health. They may have limited interest in financing healthcare (especially where there is extensive external funding) compared to other pressing budget needs such as for education, poverty eradication and infrastructure.

Academic institutions often lack the expertise to educate new professionals in relevant disciplines such as health economics, mathematical modelling, demography and population epidemiology (burden of disease), evidence-based medicine, HTA, health policy, health systems, health services research, and population-level bioethics.

## Evidence Supporting Assumptions Shaping the Theory of Change

Vogel and Breuer et al also recommend looking at evidence supporting the assumptions and contextual factors shaping the Theory of Change. I have looked at a few studies that identify challenges and pitfalls that are necessary to understand before attempts at improving priority setting decisions and processes.^28-38^ Several qualitative studies are of particular interest for understanding why there are barriers to implementing evidence from economic evaluations.

A study from Thailand by Teerawattananon and Russell reports qualitative findings on policymakers’ perspectives on the acceptability of using economic evaluation for the development of health-care benefit packages in Thailand.^31^ Among other findings, the study reported that policy actors thought that economic evaluations were relevant for decision-making because of the increasing need for rationing and more transparent criteria for making UHC decisions. They also reported a range of other factors that influenced the inclusion or exclusion of interventions: number of patients that need to be treated, severity of disease, cost of interventions and affordability, equity of access, and coverage of similar services by other health insurance schemes. Moreover, respondents raised several difficulties with using economic evaluation that would pose barriers to its introduction, including distrust in the methods used, lack of understanding of key outcomes reported (such as quality-adjusted life years [QALYs] gained and disability-adjusted life years [DALYs] averted), as well as conflicting philosophical positions and priorities compared to that of “health maximization.” Other criteria also thought to be relevant included severity of disease, social solidarity, and equity. The respondents also pointed to organizational allegiances, existing decision-making procedures that would be hard to change, and concerns about political pressure and acceptability.

A small qualitative study by Jain et al on advancing HTA in India found that although there is a good understanding of HTA among key actors, and a positive perception about producing and using HTA for decision-making among all the stakeholders interviewed, there was lack of knowledge about the subject among policy-makers at the lower levels. Importantly, at the national level, institutions prefer to treat the use of HTA evidence with caution because the capacity for adopting evidence-based tools in the health system is very limited.^39^

In a study from more than ten years ago, Kapiriri and Bondy explored health practitioners’ and health planners’ information needs and seeking behaviour for decision-making in Uganda.^40^ They reported that most of the epidemiological information was available and of relatively good quality but there was lack of information about distribution of benefits, segregated demographic data, and social values. The most often used sources of information included discussions with colleagues, doctors’ statements, and text books. They concluded that health planners and practitioners lack some of the information relevant for decision-making. Although an old study, in my experience these findings also ring true for many places today. Transferability of evidence from one context to another is also a challenge that should not be underestimated.^41^ Decision-makers often emphasise contextualised studies with local data.

In another survey of stakeholders’ values with respect to priority setting in Uganda, Kapiriri and Norheim found that respondents assign high weight to criteria such as severity of disease, benefit of the intervention, cost of the intervention, cost-effectiveness of the intervention, quality of the data on effectiveness, patient age, place of residence, lifestyle. They also emphasised the importance of providing equity of access to healthcare and being perceptive to the community’s views.^42^ These results are similar to the findings from Thailand.

In summary, in different countries, key actors report that capacity is low, access to information difficult, and understanding and knowledge about economic evaluation, HTA, and the methods used are limited. There are practical and political constraints, and there are local and national values that sometimes conflict with what is seen as a simplistic health maximizing view on what priority setting in health entails.

That said, the contextual barriers can be overcome through sensitive responses to appropriate and relevant objections. Local values matter. Moreover, the indirect influence of evidence should not be underestimated. In the last two decades, we have seen unprecedented improvements in health outcomes, partly driven by general economic growth and development, but also, I believe, through clear priorities influenced by the Millennium Developments Goals and identification of cost-effective interventions targeting infectious disease, child and maternal health among the poor. Here, DCP1 and DCP2 has also played a role, as well as international actors such as the WHO through WHO-CHOICE, the Global Fund and Gavi. Countries have made many of the right choices, and those are not incompatible with the messages based on evidence from the Disease Control Priorities project.

## Beneficiaries, Target Audience, Actors, and Sphere of Influence

The beneficiaries of better priority setting would be citizens and patients, particularly the least well off, but also health planners, academic institutions and external funders. Moving from a system of ad hoc decisions in a fragmented health sector towards more systematic, evidence based health priorities would benefit everyone. This would be, in my view, the underlying motivating factor and could guide strategic choices in a Theory of Change.

The target audiences for translating DCP evidence to better policies would include health policy makers within and outside of the MoH, health professionals, the ministry of finance, patients and patient organizations, civil society and citizens. The actors in each country would be many of the same, such as politicians, MoH officials, public health institutes, regulatory bodies, associations of medical professions, universities, health professionals, and patient organizations, but also non-governmental organizations, international donors, and regional and national World Bank and WHO offices. Among these actors, the sphere of influence in many low-income countries and lower-middle-income countries would be strongest for people working in the ministries, parliamentarians, health professions and especially doctors, and external funders. Those with weakest influence are probably patients, civil society, women, people living with stigma, and other disadvantaged groups. Among institutions, the MoH is often weaker than the ministry of finance when it comes to budgeting decisions.

## Timeline

In any Theory of Change, the chosen timeline is important. In the context of better priority setting for achieving the SDGs for health, and UHC in particular, the most appropriate timeline is the period up to 2030 – which is now little more than 10 years. Another possible timeline for countries that typically develop national strategic 5-year plans would be the time period leading up to completion of these plans. In my view, a 5-year horizon would be a minimum to achieve desired short- and perhaps long-term outputs and outcomes.

## Process Indicators

A Theory of Change also needs indicators for measuring success in achieving country led, improved priority setting in the context of UHC. Health outcomes, coverage indicators, and indicators of financial risk protections are highly relevant for the final outcomes, but probably difficult to include as indicators in a Theory of Change for translating DCP evidence into policy as discussed here. Factors interact, and other determinants beyond what priority setting can influence will also affect outcomes. Process indicators may also be relevant and appropriate. Priority-setting processes are hard to measure quantitatively, but a set of qualitative indicators can be useful. The assumption is that careful priority setting is crucial for fair progressive realization of UHC. WHO’s Consultative Group on Equity and UHC proposed the following four sets of process indicators:

- Publicly available descriptions of essential packages or identified high-priority services to which people are entitled and information about how these services are to be financed (with special emphasis on out-of-pocket payments);
- Establishment of an institution or entity within an institution (such as within the MoH) responsible for assessing and evaluating scientific evidence relevant for priority setting;
- Publicly available and well communicated criteria for priority setting;
- Establishment of decision-making bodies (such as a national priority setting commission that many northern European countries have established) that involve citizens and key stakeholders in priority setting and provide reasons for priority-setting decisions.^16^

Since better priority setting is a responsibility for countries, not DCP, the above process indicators would go beyond what DCP should be measured by. I therefore suggest the following process indicators of achieved medium- and long-term outcomes:

- Use of evidence such as that from DCP by policy makers to make priority setting decisions.
- Use of evidence such as that from DCP and explicit priority setting in strategic plans to achieve UHC or as part of national plans for primary care, NCDs, injury prevention and treatment, emergency care, surgery, or essential drugs list.

## Tentative Conclusion: Strategic Choices for Translating DCP Evidence Into Better Priority Setting

Developing a Theory of Change for a given country would include many of the elements identified above, but would be more country specific, and relevant stakeholders should be included in the process of its development. In addition, when all the elements are identified, strategic choices have to made with respect to how best to secure what needs to be in place for change to occur, how to overcome barriers, and how to reach the defined short- and long-terms goals. I shall conclude by tentatively suggesting 10 strategic choices that could be part of a Theory of Change for better priority setting:

1. Secure technical and political support for embarking on the process. Establish the value framework as is appropriate to the country in question. Goals are often available in previous national health policy documents, and will typically reflect values such as improving health, fair distribution (equity), financial risk protection, and others.
2. Develop analytic, research, planning, and process capacity within the health policy bureaucracy and management as well as in academic institutions. This will enable people to understand, demand, access, use, and translate evidence relevant for priority setting. Developing this capacity will require long-term commitments from academic institutions.
3. Make evidence easily accessible and bring it to countries.
4. Explore methods and checklists for transferring evidence from one context to another. Prioritize collection of local data and contextualized analysis.
5. Establish open, transparent processes – and if possible institutions or a standing commission – for developing country specific priority setting recommendations.
6. Include user-participation (patient organizations, topic experts, and decision-makers) in all phases leading up to recommendations, including identification of policy choices, policy space, fiscal space, evidence gaps, evidence collection, analysis, and appraisal.
7. Allow for open hearings around hard choices with key stakeholder and make the underlying rationale explicit and clear in a language that can be understood by everyone.
8. Publish recommendations in the form of nationally relevant essential packages or lists of high priority services so they are or can be known by all policy makers, actors in the health system, patients and citizens.
9. Measure key process indicators at a predefined point in time.
10. Evaluate and revise both outputs and process.
11. The evidence generated by the Disease Control Project could help countries move towards UHC and help in achieving the SDGs for health, poverty reduction and equality. Yet, it is not enough to publish large volumes and papers in high impact journals. Now the real work must begin.

## Ethical issues

Not applicable.

## Competing interests

Author declares that he has no competing interests.

## Author’s contribution

OFN is the single author of the paper.
